# Investigation of Probiotic Properties of *Lacticaseibacillus casei* 4 N‐6 Strain Isolated From Cow Milk

**DOI:** 10.1002/fsn3.70205

**Published:** 2025-04-24

**Authors:** Neslihan Dikbaş, Yusuf Can Orman, Sevda Uçar, Şeyma Alım

**Affiliations:** ^1^ Department of Agricultural Biotechnology, Agricultural Faculty Ataturk University Erzurum Turkey; ^2^ Department of Field Crops, Faculty of Agricultural Sciences and Technology Sivas Science and Technology University Sivas Turkey

**Keywords:** *Lacticaseibacillus*, *Lb*. *casei*, phytase, probiotic properties

## Abstract

The aim of the present study was to characterize the probiotic potential of *Lacticaseibacillus casei* 4 N‐6 strain isolated from cow's milk. For this purpose, acid, bile salt, pancreatin, pepsin, phenol, and lysozyme tolerance, co‐aggregation and auto‐aggregation properties, phytase production, antibiotic resistance, and antibacterial properties were analyzed in vitro. The strain had relatively poor acid, bile salt, and pepsin tolerance. However, the strain showed a high pancreatin, lysozyme, and phenol tolerance. In addition, it exhibited moderate co‐aggregation with *E. coli* and good autoaggregation. Furthermore, the cell‐free supernatant of *Lb*. *casei* 4 N‐6 showed a high antimicrobial activity against *Bacillus cereus* (N32), *Salmonella enteritidis* (RK‐485), and *Enterococcus faecalis* (RK‐487). 4 N‐6 was resistant to vancomycin, teicoplanin, gentamicin, and ceflazidime. The 4 N‐6 strain did not show hemolytic activity. In addition, this strain was found to be able to produce phytase. All the findings obtained indicate that *Lb*. *casei* 4 N‐6 is promising as a potential probiotic candidate and has superior properties that can be evaluated as a probiotic in the future. However, further research and in vivo studies are needed to fully understand its mechanism of action and optimize its use as a probiotic.

## Graphical abstract

Strain 4 N‐6 showed high pancreatin, lysozyme, and phenol tolerance. *Lb. casei* 4 N‐6 showed high antimicrobial activity against *S. enteritidis* (RK‐485), *B. cereus* (N32) and *E. faecalis* (RK‐487). The phytase activity of strain 4 N‐6 was 243.6 U/mL. Lb. casei 4 N‐6 can be used as a potential probiotic.

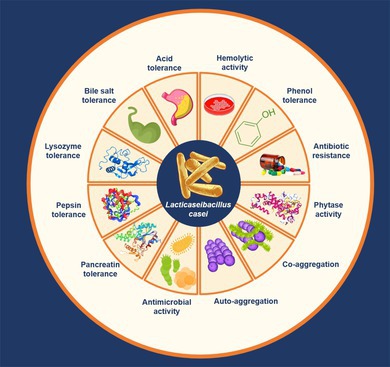

## 1 Introduction

Probiotics are generally defined as live microorganisms that provide health benefits when administered in adequate doses. They have been attracting attention for a long time due to their increasing value in industrial use and their potential benefits to human health (Das et al. 2022). Potential benefits of probiotics include lowering serum cholesterol, improving lactose metabolism, preventing mucosal infections by interfering with the colonization of pathogenic bacteria, maintaining the essential balance of the gut microbiota, and boosting immunity (Harsh and Sangita 2022).

Lactic acid bacteria (LAB) are the most favored probiotic candidates due to their enormous genetic, metabolic, and ecological diversity, having different industrial applications and being recognized as safe (GRAS) (Khushboo et al. 2023). Within this group, especially *Lactobacillus* and *Bifidobacterium* are the most studied probiotic organisms (Sornsenee et al. 2021). Lactobacillus is a Gram (+), catalase (−), aerotolerant, rod‐shaped, non‐spore‐forming, and non‐pathogenic genus (Mokoena et al. 2021). This genus is abundant in a variety of habitats, especially in carbohydrate‐rich substrates such as the human and animal gut, plants or other plant‐derived material, fermented milk, spilled food, and sewage (Naiel et al. 2021). The most commonly used *Lactobacillus* species as probiotics include *L. brevis, L. delbrueckii subsp. bulgaricus, L. delbrueckii subsp. lactis, L. paracasei subsp. paracasei, L. helveticus, L. rhamnosus, L. johnsonii, L. gasseri, L. acidophilus, L. casei, L. reuteri, L. plantarum*, and *L. fermentum* (Dempsey and Corr 2022). However, not all strains exhibit probiotic properties due to strain‐specific characteristics. Therefore, the identification and selection of strains to be used as potential probiotics is very important to increase the chances of obtaining competent probiotic strains (Torres‐Maravilla et al. 2022).

Commonly stated selection criteria for probiotic strains include being tolerant to acids and bile, non‐carcinogenic and non‐pathogenic, adhering to and colonizing the host epithelial tissue, enriching the intestinal microflora, reducing pathogenic adhesion, and producing secondary metabolites antagonistic to pathogens (Terpou et al. 2019; Sornsenee et al. 2022). Candidate strains need to be thoroughly tested for safety and probiotic properties. In the present study, it was aimed to determine the probiotic potential of *Lb*. *casei* 4 N‐6 isolated from village milk. Therefore, the strain's antimicrobial activity, tolerance to gastrointestinal tract conditions, adherence to epithelial cells, and some safety‐related probiotic properties were evaluated by a series of in vitro tests.

## 2 Materials and Methods

### 2.1 Bacterial Strain

*Lb. casei* strain 4 N‐6 was isolated from cow milk; the PCR product was sent for sequencing, and the sequences obtained were identified by BLAST analysis in the NCBI GenBank database. According to the BLAST results, the sequence obtained showed the highest similarity of 100% with the *Lacticaseibacillus casei* strain, and the results were registered in NCBI (GenBank accession number: ON261567). The strain is stored in N. DİKBAŞ culture collection at Atatürk University, Faculty of Agriculture, Department of Agricultural Biotechnology at −80°C under the code 4 N‐6. Pathogenic strains ( *B. cereus* (N32), *Pseudomonas aeruginosa* (RK‐481), *S. enteritidis* (RK‐485), *E. faecalis* (RK‐487) and *Staphylococcus aureus* (RK‐484)) are stored at −80°C in the culture collection of the Department of Plant Protection, Faculty of Agriculture, Atatürk University.

### 2.2 Acid (Low pH) Tolerance

The acid tolerance of strain 4 N‐6 was determined using the method of Dikbaş et al. (2024). The density of the bacterial suspension was adjusted by measuring the absorbance at 600 nm using 0.5 McFarland's solution (OD600: 0.08–0.10). The active culture was inoculated (1%) into MRS broth with different pH values (pH 2, 2.5, 3, and 7.0 (control)) and kept at 37°C for 3 h. Then, a tenfold dilution was made using 0.01 M PBS buffer (pH 6.2). Samples were spread on MRS agar and incubated at 37°C for 36 h. The viability rate of the strain was determined using the formula below (Equation 1).

(1) $$\text{Survival rate of strain}\left(\%\right)=\frac{\log\mathrm{CFU}\;N1}{\log\mathrm{CFU}\;N0}\times 100$$

N1 refers to treatment application and N0 refers to control.

### 2.3 Bile Salt Tolerance

The bile salt tolerance of the strain was examined following the method used by Hajikhani et al. (2021). Active culture (0.5 McFarland) was inoculated (1%) into MRS broth medium containing different concentrations of oxgall (1.5%, 1.0%, 0.5%, 0.3% and 0.0% w/v) and kept at 37°C for 3 h. After incubation, samples were transferred to MRS agar by tenfold dilution in 0.01 M PBS buffer (pH 6.2) and incubated (36 h at 37°C). The survival rate of the strain was then evaluated (Equation 1).

### 2.4 Pepsin Tolerance

Pepsin resistance of the strain was evaluated by adding 3 mg/mL pepsin to PBS buffer with different pH values. The active culture (0.5 McFarland) was inoculated (1%) into the prepared PBS buffers and kept at 37°C for 3 h (Simsek et al. 2022). The viability of the strain was determined at the beginning of the incubation, at 1 and 3 h (Equation 1).

### 2.5 Pancreatin Tolerance

Pancreatin tolerance of the strain was determined by inoculating the active culture (0.5 McFarland; 1%) into PBS buffer (pH 8.0) containing 1 mg/mL pancreatin (pH 8.0) and incubating at 37°C for 4 h (Simsek et al. 2022). The viability of the strain was determined at the beginning and end of the 4th hour (Equation 1).

### 2.6 Lysozyme Tolerance

The lysozyme tolerance of the strain was determined by inoculating 1% of the active culture (0.5 McFarland) into MRS Broth (10 mL) with and without 100 mg/L lysozyme and kept at 37°C for 90 min (Surono 2003; Sakurai et al. 2017; Tarique et al. 2022). The viability of the strain was determined at the beginning and at 90 min (Equation 1).

### 2.7 Phenol Tolerance

Phenol tolerance of *Lb*. *casei* 4 N‐6 was evaluated by modifying the method used by Adithi et al. (2022). The active culture (0.5 McFarland; 1%) was inoculated into MRS Broth containing 0.3% phenol and incubated at 37°C for 24 h. The viability of the strain was determined at the beginning and end of the incubation.

### 2.8 Antibiotic Resistance

Antibiotic susceptibility of the strain was tested against 13 different antibiotics (trimethoprim (5 μg/disc), rifampin (5 μg/disc), penicillin G (6 μg/disc), bacitracin (10 μg/disc), ampicillin (10 μg/disc), meropenem (10 μg/disc), gentamicin (10 μg/disc), erythromycin (15 μg/disc), ceftazidime (10 μg/disc), teicoplanin (30 μg/disc), tetracycline (30 μg/disc), vancomycin (30 μg/disc) and piperacillin (100 μg/disc)). From the active cultures (0.5 McFarland), 100 μL of MRS agar was spread on petri dishes. Antibiotic discs were then placed in the center of these plates and incubated for 24 h (at 37°C; Tilwani et al. 2022). The clear zone diameter (mm) formed around the disc was measured and evaluated according to CLSI (Coelho‐Rocha et al. 2023; Haley et al. 2024).

### 2.9 Antimicrobial Activity

The antimicrobial effect of *Lb*. *casei* 4 N‐6 against five pathogens ( *S. enteritidis* (RK‐485), *P. aeruginosa* (RK‐481), *B. cereus* (N32), *E. faecalis* (RK‐487) and *S. aureus* (RK‐484)) was evaluated using the agar‐well diffusion method (Rocha‐Ramírez et al. 2021). The strain incubated in MRS broth at 37°C for 24 h was centrifuged at 5000 rpm for 15 min, and the supernatant was subjected to microfiltration (0.45 μm). In Mueller Hinton Agar containing pathogenic strains, wells (6 mm diameter) were made with a swab, and 100 μL of supernatant was transferred. After incubation (24 h), the zone diameter (mm) formed around the well was measured.

### 2.10 Auto‐Aggregation and Co‐Aggregation

The auto‐aggregation property of strain 4 N‐6 was evaluated using the method used by Nami et al. (2019) with minor modifications. The percentage of auto‐aggregation was calculated according to the formula given below as a result of the measurements made at the end of the initial, 2nd, and 5th hours.

$$\text{Auto}-\text{aggregation}=\left(1-\frac{\mathrm{At}\;\left(\text{Absorbance}\;\mathrm{at}\;\text{time}\;t\right)}{A0\;\left(\text{Represents absorbance}\;\mathrm{at}\;0\right)}\right)\times 100$$

The co‐aggregation property of the strain with *E. coli* was determined according to the method used in the study of Yadav et al. (2017) with minor changes after incubation at 37°C for 4 h using the following formula.

$$\mathrm{Co}-\text{aggregation}\;\left(\%\right)=\left(\frac{\left(\mathrm{OD}1+\mathrm{OD}2\right)-2\left(\mathrm{OD}3\right)}{\left(\mathrm{OD}1+\mathrm{OD}2\right)}\right)\times 100$$

OD1: optical density of *Lb*. *casei* 4 N‐6, OD2: optical density of *E. coli* , OD3: optical density of a mixture of *Lb*. *casei* 4 N‐6 and *E. coli* after 4 h.

### 2.11 Phytase Enzyme Activity

0.1 mL phytase‐containing bacterial supernatant was mixed with 0.25 mL sodium phytate (2 mM) and kept at 50°C for 10 min. Then 10% trichloroacetic acid (TCA) (0.5 mL) was added, and the reaction was allowed to finish for 15 min. The absorbance of the enzyme at 700 nm was measured against the control (0.1 mL distilled water +0.25 mL sodium phytate +0.5 mL TCA (10%); Demir et al. 2018).

### 2.12 Hemolytic Activity

Hemolytic activity was performed as described by Ahire et al. (2021). An overnight culture of *Lb. casei* 4 N‐6 grown on MRS agar was inoculated onto blood agar plates (containing 7% (v/v) sheep blood) and incubated at 37°C for 48 h. After incubation, the plates were observed for α‐hemolysis (dark and greenish zones), β‐hemolysis (light yellow or transparent zones) and γ‐hemolysis (no change or no zones).

### 2.13 Statistical Analysis

Statistical analysis of the data was performed using SPSS (version 20.0) software (SPSS Inc., Chicago, IL, USA). Comparison between means was determined by HSD Tukey test and independent sample *t* test. All experiments were performed in triplicate. Results were expressed as mean ± SD.

## 3 Results and Discussion

**FIGURE 1:**
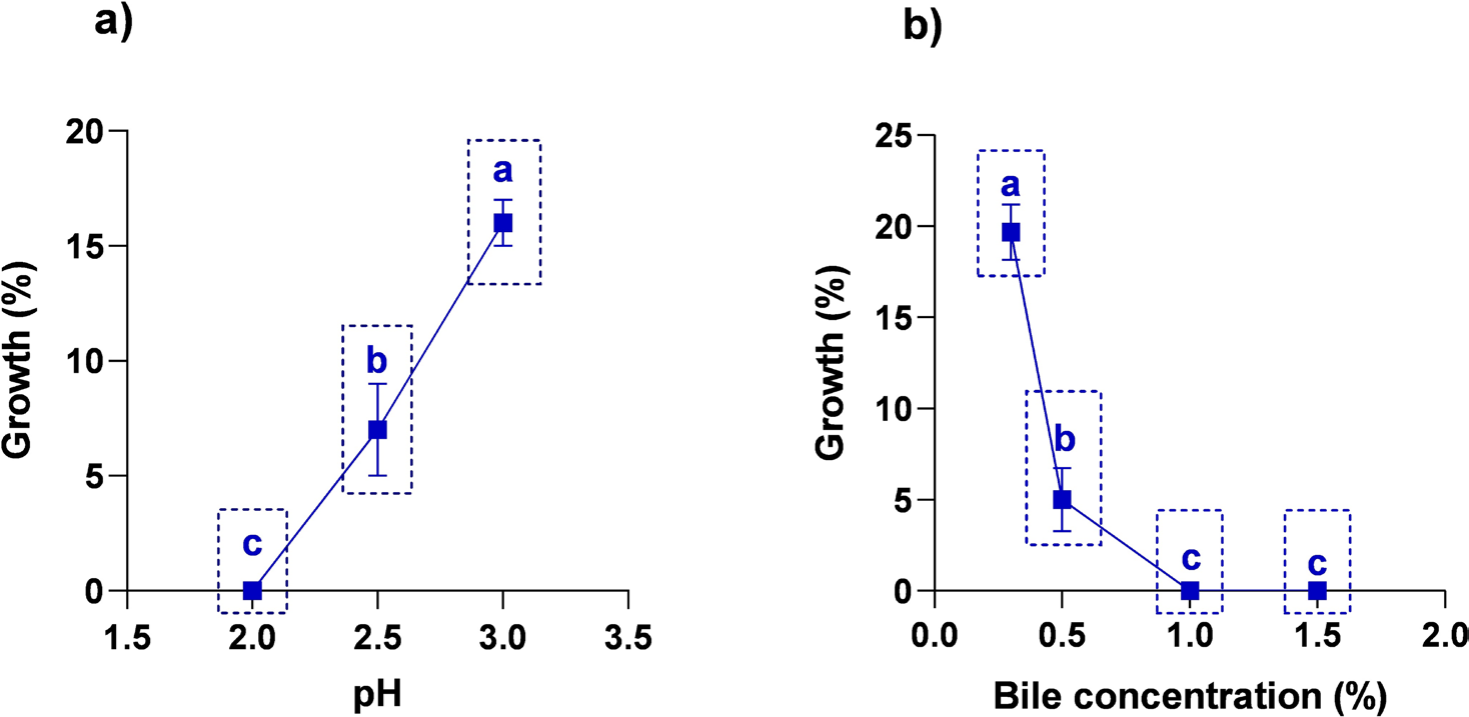
Acid (a) and bile (b) resistance profiles of *L. casei* 4 N‐6. Values presented represent the mean ± standard deviation of three replicate runs. Different letters on the graph indicate significant differences between groups (*p* < 0.05).

The identification of autochthonous LAB with probiotic properties paves the way for the emergence of new isolates that are probiotic candidates (de Souza da Motta et al. 2022). The results obtained in this study add information to the few data available in the literature on the probiotic properties of *L. casei* . Acid tolerance, one of the probiotic properties, is a critical factor in the survival and functionality of bacteria in gastric juice (Liu et al. 2020). It affects their ability to survive in the gastrointestinal (GI) system and in various industrial applications. Therefore, the investigation of acid tolerance of probiotics is very important for their development and application (Szutowska and Gwiazdowska 2021). Acid tolerance of *Lb*. *casei* 4 N‐6 strain was tested at pH 2.0, 2.5, and 3.0, and its growth at pH 7.0 was considered as control. The results obtained were statistically analyzed, and the differences between the groups were found significant according to the HSD Tukey multiple comparison test result (*p* < 0.05; Figure 1a). *Lb*. *casei* 4 N‐6 showed no viability at pH 2.0, 7% at pH 2.5, and 16% at pH 3.0 after 3 h. In contrast, 12 *L. casei* strains isolated from mozzarella cheese maintained viability at pH 2.0 for 2 h (de Souza et al. 2019). Similarly, *L. casei* MYSRD 108 isolated from fermented foods showed a survival rate of over 70% at both pH 2.0 and pH 4.0 (Divyashree et al. 2021). Related studies (Yusuf et al. 2020; Sharma et al. 2021) and results show that the acid tolerance profile is strain specific. The low acid tolerance of *Lb*. *casei* 4 N‐6 is a major limitation to its ability to survive in the gastrointestinal tract. However, to alleviate this limitation, various technological approaches have been developed to increase the acid resistance of probiotics. These approaches are envisioned to increase the survival ability of the strain and potentiate probiotics (Xu et al. 2022; Barajas‐Álvarez et al. 2023).

Bile salt tolerance is one of the most important criteria in the selection of probiotic strains as it affects the survival of bacteria in the small intestine. Bile salts show antimicrobial effects by disrupting the cell membrane (phospholipids and surface proteins) and intracellular homeostasis (Chen et al. 2022). The physiological concentration of bile in humans is around 0.3%. Therefore, this concentration is considered a critical limit during probiotic bacteria screening (Divyashree et al. 2021; Nath et al. 2020). Bile tolerance was determined after 3 h incubation of the strain in liquid cultures with different (0.3%, 0.5%, 1.0%, 1.5%) bile salt concentrations (Figure 1b). The results were evaluated by the HSD Tukey multiple comparison test and a significant relationship between bile concentration and viability was revealed (*p* < 0.05). *Lb*. *casei* 4 N‐6 showed a survival of 19.6% and 5% after 3 h exposure to 0.3% and 0.5% bile salt concentrations, respectively, and the difference between the survival rates was significant (*p* < 0.05; Figure 1b). The strain did not show viability at high bile concentrations (1.0% and 1.5%). *L. casei* C3 (Reuben et al. 2020) and *L. casei* MYSRD 108 (Divyashree et al. 2021) strains isolated from cow's milk and fermented foods showed a better tolerance to 0.3% bile concentration than the *Lb*. *casei* 4 N‐6 strain. In addition, it is also seen in studies that the increase in bile concentration causes a decrease in the viability rates of strains (Liu et al. 2020; Dikbaş et al. 2024; Jia et al. 2024). Considering the results obtained, it is predicted that the strain 4 N‐6 can survive at low bile salt concentrations in the small intestine.

Pancreatin and pepsin tolerance is a very important criterion for the survival and functionality of probiotic bacteria, especially in the gastrointestinal tract (Akmal et al. 2022). These enzymes are essential components of digestive fluids, and their presence can significantly affect the survival and growth of probiotic bacteria (Mohammad et al. 2020). Tolerance to digestive enzymes allows the bacteria to maintain their functionality and thus contribute to the overall health of the host (Wendel 2022).

**FIGURE 2:**
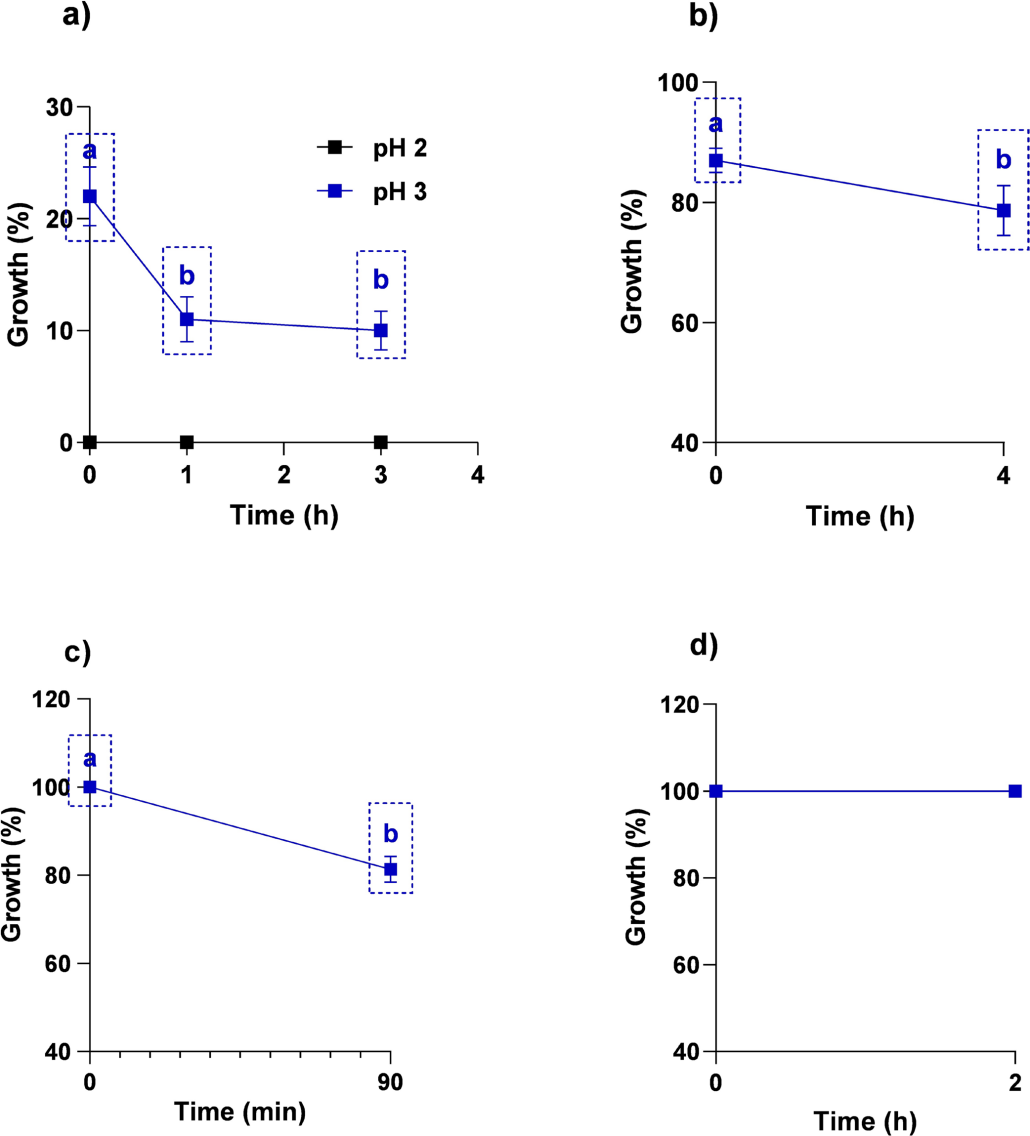
Pepsin (a), pancreatin (b), lysozyme (c) and phenol (d) resistance profile of *L. casei* 4 N‐6. Values presented represent the mean ± standard deviation of three replicate runs. Different letters on the graph indicate significant differences between groups (*p* < 0.05).

The pepsin tolerance of *Lb*. *casei* 4 N‐6 was determined at pH 2.0 and 3.0 at the end of the initial, 1st, and 3rd hours. The results were evaluated statistically, and the differences between the groups were found significant according to the HSD Tukey multiple comparison test (*p* < 0.05). The strain showed no viability at pH 2.0 in pepsin medium, while it showed 22%, 11%, and 10% viability at pH 3.0 at the end of 0, 1, and 3 h, respectively (Figure 2a). The viability rate of the strain decreased with time, and the difference between the viability rate at the beginning (0 h) and at the other hours (1 and 3 h) was found to be significant (*p* < 0.05). Pancreatin tolerance was determined at pH 8.0 at 0 h and at the end of 4 h. The results were statistically analyzed (independent sample t test) and the differences were found to be significant (*p* < 0.05). Strain 4 N‐6 exhibited good tolerance to pancreatin at pH 8, with a viability of 87% and 79% at baseline and at the end of 4 h, respectively (Figure 2b). *L. casei* NM512 and *L. casei* ATCC 393 showed a similar tolerance profile to pancreatin (Mansour et al. 2014; Soltani et al. 2022). The pepsin and pancreatin resistance of 11 LAB isolated from traditional fermented foods and beverages of Ethiopia was investigated, and it was reported that the survival rate of all isolates tested ranged from 56.37% to 88.21% for pepsin and 56.14%–106.50% for pancreatin (Amenu and Bacha 2023). *Lb*. *casei* 4 N‐6 strain showed a lower pepsin tolerance than different *Lactobacillus* species in the literature, while it showed parallel results in terms of pancreatin tolerance (Amenu and Bacha 2023; Ben Farhat et al. 2022).

Lysozyme tolerance is a very important factor for probiotics, especially those designed for oral administration because lysozyme in saliva is the first obstacle that probiotics encounter in the oral environment. The ability of a probiotic to tolerate lysozyme is very important for its survival and functionality (Ahn et al. 2023). In order to test lysozyme tolerance, *Lb*. *casei* 4 N‐6 strain was exposed to lysozyme (100 mg/L) for 90 min, and the results obtained were evaluated by independent sample *t* test. While the strain showed 100% viability at the beginning in lysozyme medium, it showed 81.3% viability at the end of 90 min, and the difference between the two groups was significant (*p* < 0.05; Figure 2c). The viability decreased proportionally with time. The results are consistent with lysozyme tolerance profiles of LAB isolated from different plant leaves, human fecal samples, and breast milk (Samedi and Charles 2019; Duraisamy et al. 2022; Debnath et al. 2023). Considering the data obtained, it is seen that *Lb*. *casei* 4 N‐6 strain shows a high tolerance to lysozyme.

Phenol is a toxic metabolite secreted by pathogens in the gut during the deamination of certain amino acids and can exert bactericidal effects against LAB. Therefore, phenol tolerance is a critical trait for probiotic bacteria, and testing of candidate strains for phenol resistance is mandatory (Dikbaş et al. 2024). In the study, phenol tolerance of *Lb*. *casei* 4 N‐6' strain was determined after exposure to 0.3% phenol concentration for 48 h. At the 48th hour, no decrease was observed in viable bacterial counts. The strain exhibited a very good tolerance to phenol with 100% viability at baseline and 48 h (Figure 2d). Many studies support that *Lactobacillus* spp. exhibit a high resistance to phenol (Fonseca et al. 2021; Soliman et al. 2021). Similarly, *L. casei* C3, *L. casei* RAMULAB07, and *L. casei* RAMULAB08 strains isolated from different sources were reported to tolerate 0.4% phenol (Reuben et al. 2020; Kumari et al. 2022). The high phenol tolerance of *Lb*. *casei* 4 N‐6 strain will allow it to survive its passage through the gastrointestinal tract.

Antibiotic sensitivity should be considered as an important part of safety assessment for the evaluation of probiotics. LAB are mostly considered safe, but their safety characteristics should be evaluated before administration (Daliri et al. 2022; Megur et al. 2023). In the present study, the resistance of the strain against 13 different antibiotics was determined, and the findings are given in Table 1. The strain was resistant to vancomycin (6.0 ± 0.0 mm), teicoplanin (6.3 ± 0.6 mm), gentamicin (7.3 ± 0.6 mm) and ceflazidime (8.7 ± 3.1 mm), respectively. Regarding other antibiotics, it was observed that it was moderately resistant to bacitracin (19.3 ± 0.6 mm), susceptible to tetracycline, a broad spectrum antibiotic, and other antibiotics listed in Table 1. Similarly, de Souza et al. (2019) reported that 12 *L. casei* strains were resistant to vancomycin and sensitive to tetracycline. However, unlike the current study, they stated that all strains were moderately sensitive to gentamicin. Studies in the literature report that *Lactobacillus* has intrinsic resistance to aminoglycosides streptomycin and gentamicin and glycopeptides vancomycin and teicoplanin (Reuben et al. 2019; Li et al. 2020). The main feature of the intrinsic resistance mechanism in *Lactobacillus* is that these bacteria can survive in the presence of antibiotics without the transfer of resistance genes to other microorganisms (Das et al. 2020; Szutowska and Gwiazdowska 2021). It has been reported that the natural resistance of *Lactobacillus* to various antibiotics may allow the strains to remain in the intestinal microflora for a longer period of time and to be used simultaneously with the antibiotic to which they are resistant (Nath et al. 2020; Hassan et al. 2020).

**TABLE 1:** Antibiotic susceptibility profile of *L. casei* 4 N‐6.

Antibiotic	Zone diameter (mm) ± SD^a^	Antibiotic susceptibility profile^b^
Vancomycin (30 μg)	6.0 ± 0.0	R
Teicoplanin (30 μg)	6.3 ± 0.6	R
Gentamicin (10 μg)	7.3 ± 0.6	R
Ceflazidime (10 μg)	8.7 ± 3.1	R
Bacitracin (10 unit)	19.3 ± 0.6	I
Penicillin (10 unit)	26.3 ± 1.5	S
Ampicillin (10 μg)	29.0 ± 1.7	S
Meropenem (10 μg)	30.0 ± 1.0	S
Trimethoprim (5 μg)	34.7 ± 2.1	S
Erythromycin (15 μg)	36.7 ± 3.1	S
Tetracycline (30 μg)	38.0 ± 1.0	S
Rifampin (5 μg)	44.0 ± 1.0	S
Piperacilin (100 μg)	44.3 ± 2.9	S

^a^ Data represent the mean ± standard deviation of three replicate runs.

^b^ I, intermediate; R, resistant; S, sensitive.

One of the characteristics sought in probiotic candidates is the presence of antimicrobial activity (Echegaray et al. 2023). The antimicrobial or antagonistic property of probiotics involves the synthesis of antimicrobial compounds, the enhancement of intestinal barrier function in resisting pathogens, and the strengthening of the host immune system to successfully combat pathogens (Silva et al. 2020; Fijan 2023). LAB are known to inhibit the growth of pathogens through the production of antimicrobials such as bacteriocins, organic acids (acetic acid and lactic acid) and hydrogen peroxide (Mendoza et al. 2023). In the study, the antimicrobial activity of *Lb*. *casei* 4 N‐6 was tested against five pathogenic bacteria and showed an inhibitory effect against all pathogens except *P. aeruginosa* (RK‐481). The strain formed a clear zone of inhibition diameter that was strong against *E. faecalis* (RK‐487), *B. cereus* (N32), *S. enteritidis* (RK‐485) and weak against *S. aureus* (RK‐484; Table 2). In parallel with the results of the present study (Xu et al. 2020), it showed that *L. casei* NA‐2 could inhibit the growth of *B. cereus* , *Escherichia coli* O157:H7, *Salmonella typhimurium* , and *S. aureus* . In addition, Divyashree et al. (2021) reported that *L. casei* MYSRD 108 has antibacterial activity against *E. coli* (ATCC 25,922), *S. aureus* (ACTT 6538), *K. pneumoniae* (MTCC 7407) and *P. aeruginosa* (ATCC 15,422). Similarly, *L. casei* B22 showed antimicrobial activity against *P. aeruginosa, S. enterica serovar Typhimurium, E. coli*, and *S. aureus* (Barzegar et al. 2021).

**TABLE 2:** Antimicrobial activity of *L. casei* 4 N‐6 against five different pathogens.

Pathogenic microorganism	*L. casei* 4 N‐6^a^
*Enterococcus faecalis* (RK‐487)	+++
*Salmonella enteritidis* (RK‐485)	+++
*Bacillus cereus* (N32)	+++
*Staphylococcus aureus* (RK‐484)	+
*Pseudomonas aeruginosa* (RK‐481)	−

^a^ −: No zone; +: Zone diameter between 0 and 3 mm (weak); ++: Zone diameter between 3 and 6 mm (good); ++++: Zone diameter ≥ 6 mm (strong). The diameter of the well drilled into the agars is 6 mm.

**FIGURE 3:**
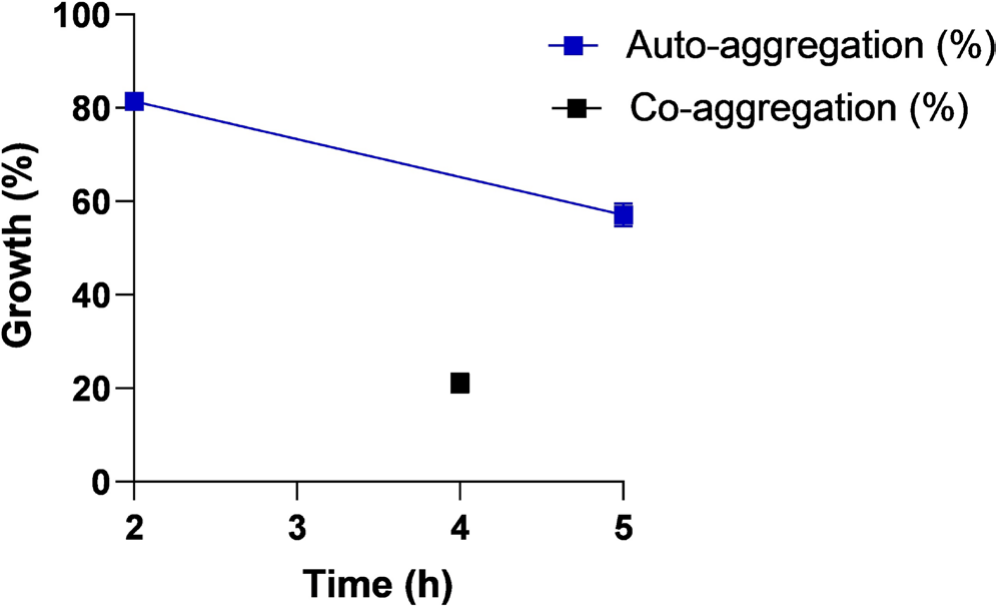
Auto‐aggregation and co‐aggregation profile of *L. casei* 4 N‐6. Values presented represent the mean ± standard deviation of three replicate runs.

Auto‐aggregation and co‐aggregation are vital properties of probiotics that contribute to their survival, colonization, and interactions with other bacteria or the host environment (Nwoko and Okeke 2021). Auto‐aggregation refers to the adhesion between cells of the same bacteria and contributes to probiotics reaching high cell concentrations in the GI tract and adhering to the intestinal epithelium. Co‐aggregation is the intercellular adhesion between different species and helps pathogenic bacteria form a barrier that prevents colonization and biofilm formation (Li et al. 2020; Barzegar et al. 2021). In the study, the percentage of auto‐aggregation of *Lb*. *casei* 4 N‐6 was determined as 81.42% and 57.14% after 2 and 5 h of incubation, respectively (Figure 3). The relationship between time and auto‐aggregation (%) was analyzed statistically (independent sample t test) and the differences were found significant (*p* < 0.05). The percentage of co‐aggregation decreased as the incubation time increased. The strain showed a co‐aggregation of 21.2% with *E. coli* (Figure 3). The *L. casei* C3 strain showed an auto‐aggregation characteristic (54.50%) in line with the study results, while it showed a higher co‐aggregation rate (96.78%) with *E. coli* (Reuben et al. 2020). *L. casei* B22 isolated from Iranian cheese showed a lower auto‐aggregation (~30%) and co‐aggregation (12.3% with *E. coli* ) than the current study strain *Lb*. *casei* 4 N‐6 (Barzegar et al. 2021).

Phytase is a very important enzyme involved in the digestive process, especially in the breakdown of phytates, a type of dietary fiber found in many plants. It can improve nutrient intake, aid digestion, reduce environmental pollution, and increase the nutritional value of foods. Therefore, it is very important for both human health and the environment that probiotic bacteria produce phytase (Bhagat et al. 2020). In this study, *Lb*. *casei* 4 N‐6 was found to have a phytase activity of 243.6 U/mL. The result obtained supports the ability of LAB to produce phytase (Sharma et al. 2020; Dikbaş et al. 2023).

**FIGURE 4:**
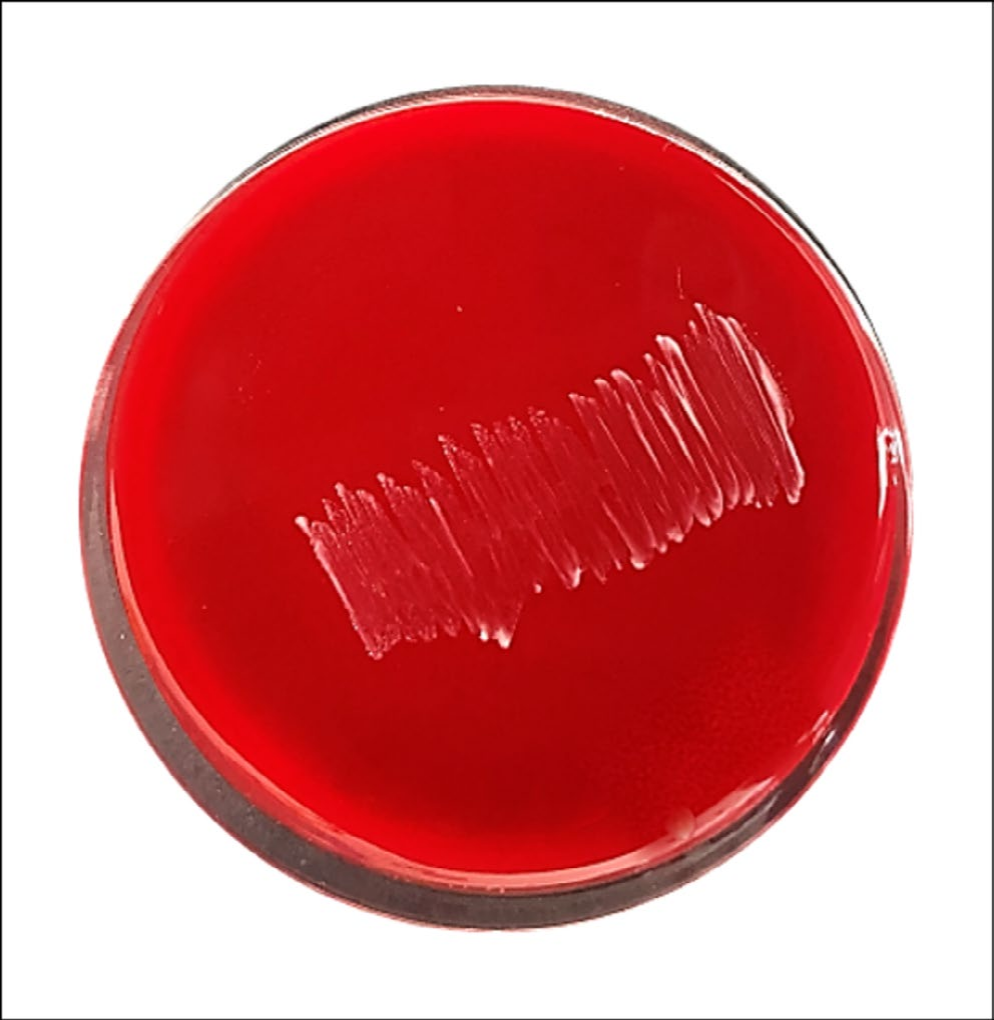
γ‐hemolytic activity of *L. casei* 4 N‐6 strain.

In vitro testing of hemolytic activity is one of the safety requirements used to evaluate potential probiotic strains (Lemos Junior et al. 2020). Hemolysis activity is undesirable in probiotic strains as they are considered virulence factors (Çetin and Aktaş 2024). *Lb. casei* strain 4 N‐6 did not exhibit hemolytic activity on blood agar plates (Figure 4). Similarly, *L. paracasei* CT12 (Romero‐Luna et al. 2020) and *Lb. casei* XN18 (Zibaei‐Rad et al. 2023) strains did not show hemolytic activity.

## 4 Conclusion

*Lb. casei* 4 N‐6 strain isolated from village milk (cow) showed moderate acid, bile salt, and pepsin tolerance and very good pancreatin, lysozyme, and phenol tolerance in vitro. In addition, strain 4 N‐6 was found to have a strong antimicrobial activity against *B. cereus* (N32), *S. enteritidis* (RK‐485) and *E. faecalis* (RK‐487). The strain exhibited good auto‐aggregation and co‐aggregation properties and phytase activity. The obtained findings indicate that *Lb*. *casei* 4 N‐6 strain has probiotic properties. With the increase in studies on *L. casei* , it is thought that this strain can be used in the production of different products in various industrial sectors. However, it is important to note that further research and in vivo studies are needed to confirm the probiotic effects of the strain and to fully understand its potential benefits and risks.

## Author Contributions

**Neslihan Dikbaş:** formal analysis (equal), funding acquisition (equal), methodology (equal), project administration (equal), writing – original draft (equal), writing – review and editing (equal). **Yusuf Can Orman:** formal analysis (equal), methodology (equal), writing – original draft (equal), writing – review and editing (equal). **Sevda Uçar:** supervision (equal), writing – original draft (equal), writing – review and editing (equal). **Şeyma Alım:** methodology (equal), writing – original draft (equal), writing – review and editing (equal).

## Ethics Statement

The authors have nothing to report.

## Conflicts of Interest

The authors declare no conflicts of interest.

## Data Availability

All data relevant to the article are included in the article.
